# Supplementary material for: Using metacommunity ecology to understand environmental metabolomes
Source: Nat Commun. 2020 Dec 11;11:6369. doi: 10.1038/s41467-020-19989-y (PMC7732844; doi:10.1038/s41467-020-19989-y)

Supplementary Information for:
Using metacommunity ecology to understand environmental metabolomes

Supplementary Legend
Supplementary Document 1: This document provides additional discussion regarding the three metabolite dendrograms.

Supplementary Table 1: A table providing coordinates for the field sites analyzed in this manuscript.

Supplementary Figure 1: Spatial distribution of sampled field sites in southeast Washington State, including photos of surficial sediments at each sampling location. The quadrant is 1m x 1m. Three independent push point samplers were used in each quadrant to collect pore water and a river water sample was collected from an adjacent location.

Supplementary Figure 2: Dendrograms and sub-dendrograms with associated metrics. Panels a, c, and e are the visualizations of the Molecular Characteristics Dendrogram (MCD), Transformation-based Dendrogram (TD), and Transformation-Weighted Characteristics Dendrogram (TWCD), respectively. Panels b, d, and f contain sub-dendrograms of the 16 most frequently observed metabolites to demonstrate the clustering patterns of elemental composition and two metrics: average transformation count and nominal oxidation state of carbon (NOSC). Average transformation count is the average number of transformations associated with a given metabolite across all of the samples. NOSC is a metric that represents the thermodynamic availability of metabolites.

Supplementary Figure 3: A bar chart depicting the number of FTICR-MS detected peaks obtained across the dataset (Total), the number peaks assigned a molecular formula (Assigned), and the number of peaks incorporated into each of the three dendrograms (MCD, TD, TWCD). The numbers above each bar correspond to the height of the bar.

Supplementary Figure 4: Histogram demonstrating the number of FTICR-MS identified peaks with a given number of associated biochemical transformations. The inset plots only those peaks with 100 or fewer transformation counts; the arrow indicates the location of the 40 count which only had 1.

Supplementary Document
Metabolite Dendrograms
In order to investigate metabolite clustering patterns on the three dendrograms, we examined a subset of the 16 most frequently occurring metabolites within the dataset (i.e., those metabolites which appeared in the most samples) (**Supplementary Figure 2**). Furthermore, we discuss the costs and benefits of each dendrogram and their potential interpretations. All dendrograms generated during this study are provided in the supplemental material (**Supplementary Data 1-3**).

Molecular Characteristics Dendrogram (MCD)
Following principles similar to those established in compound classification studies [1–3], identified metabolites can be grouped based upon their molecular characteristics. We specifically

used elemental composition (e.g., C-, H-, O-, N-, S-, P-content), double-bond equivalents (DBE), modified aromaticity index (AI_{Mod}), and Kendrick's defect, which all provide information regarding the composition and structure of metabolites (i.e., saturated vs. unsaturated, nitrogen content, etc.) [4–7]. These metrics were combined to estimate a pairwise Euclidean distance matrix. UPGMA (unweighted pair group method with arithmetic mean) hierarchical clustering was subsequently used to generate a dendrogram approximating compositional similarities across metabolites (**Supplementary Figure 2ab**). This dendrogram is referred to as the Molecular Characteristics Dendrogram (MCD).

The MCD appeared to provide reasonable and expected patterns of clustering. This is made clear by examining the sub-dendrogram of 16 of the most frequently observed metabolites across our sample set, within which discrete clusters formed based on metabolite characteristics. For example, metabolites containing O_3S (i.e., $C_{17}H_{28}O_3S$, $C_{16}H_{26}O_3S$, $C_{18}H_{30}O_3S$) clustered together, suggesting that this feature is a dominating trait (**Supplementary Figure 2b**). In addition, more complex metabolites (i.e., those containing a mixture of O, N, S, and P, like $C_9H_3N_2O_8SP$) clustered separately from simpler CHO/CHON metabolites (**Supplementary Figure 2b**).

From this initial analysis the MCD appears to provide interpretable clustering patterns that make conceptual sense. We propose that the MCD can be used in analyses that are directly analogous to phylogenetic and functional trait analyses within community ecology. An important caveat is that while straightforward to generate and interpret, the MCD requires that metabolites have assigned chemical formulas. This requirement can lead to large portions of data being dropped. For example, within this FTICR-MS dataset, ~14% of resolved metabolites (i.e., peaks in the mass spectra) were assigned a formula. Analyses based on an MCD will, therefore, exclude a significant portion of metabolites. However, we contend that this should not preclude use of MCDs as important insights are commonly revealed through analyses of metabolite subsets that have assigned formulas [8–13]. Moreover, such shortcomings can be alleviated as formula assignment algorithms improve, or through the use of other mass spectrometry techniques that can provide further biological or chemical context, like ion mobility spectrometry mass spectrometry which can detect structural information via the collision cross section measurement [14,15]

<u>Transformation-based Dendrogram (TD)</u>

To provide an approach that is complementary to the MCD and that makes use of more of the resolved metabolites, we developed a dendrogram method based on inferred biochemical transformations occurring among metabolites. This is referred to as the Transformation-based Dendrogram (TD) and is enabled by the ultrahigh mass resolution of FTICR-MS, which allows all resolved metabolites with or without assigned formulas to be organized in a putative transformation network based upon a database. This network is estimated through a series of putative biochemical transformations that are inferred through quantitative estimation of between-metabolite mass differences [2,11,12,16–18]. For example, if the mass difference between two metabolites is 18.0343, that would indicate that there is a loss or gain of an ammonium group, while a mass difference of 103.0091 would indicate loss or gain of a cysteine. However, both the reactant and product must be present for a transformation to be detected; if one is missing, no transformation will be considered. Lists of known biochemical transformations have been previously compiled and used in analyses of ecosystem metabolomes (**Supplementary Data 4**; Graham *et al.* 2018; Stegen *et al.* 2018).

To estimate the TD, we quantified the minimum number of steps a given metabolite must take to reach another metabolite in the network (e.g., metabolite 1 can become metabolite 2 via a given set of transformations). Doing so across all pairwise metabolite comparisons provides quantitative relational information among many metabolites in a given dataset (**Figure 1**). In turn, the TD is estimated from the pairwise metabolite network distances and is therefore based on information of how each metabolite could give rise to another (**Supplementary Figure 2cd**).

The topology of the TD can diverge from the MCD because it is based on putative biochemical transformations that link metabolites, rather than metabolite molecular characteristics (**Supplementary Figure 2d**). For example, in the MCD there were high O-content (i.e., $C_9H_2O_{10}$, $C_{10}H_2O_{11}$) and low O-content (i.e., $C_{17}H_{26}O_5$, $C_{18}H_{34}O_3$, etc.) clusters that diverged from each other, but were relatively near each other in the TD. This is likely because there are a small number of transformations (i.e., network steps) separating metabolites with high O-content from the low O-content metabolites. In addition, other metabolite-specific variables such as average transformation count, which measures the average number of transformations associated with a given peak, are more strongly associated with the topology of the TD than the MCD (**Supplementary Figure 2d**). This points to structure within the network whereby highly connected metabolites are linked to other highly connected metabolites through a small number of biochemical transformations. This type of non-random structure within the broader transformation network is captured in the TD and therefore informs downstream evaluation of processes governing the spatiotemporal organization of metabolite assemblages.

Importantly, the TD can be generated without formula assignments. This allows a greater number of metabolites to be used in and inform other analyses, which is an advantage over the loss of data that occurs with the MCD. While this increase in peak representation can be beneficial, it does come at the cost of not considering metabolite characteristics that provide biogeochemically-relevant stoichiometric information. Another caveat with the TD approach, however, is that the transformations are limited by the available database. While this database is broadly applicable across ecosystems, it is not exhaustive and will likely represent an under-estimation of biochemical potential. As we identify more putative biochemical transformations, these can be integrated with this existing dendrogram to enhance metabolite placement, akin to concatenated single copy gene trees revealing deeper lineages than the 16S rRNA gene in microbial communities [19–21]. Therefore, the TD and MCD should be viewed as complementary. There are likely scenarios in which analyses based on TD and MCD would be used together to deepen insights.

Transformation-Weighted Characteristics Dendrogram (TWCD)

While the MCD and TD each have unique strengths, there is an opportunity to combine these approaches to obtain more information than is available with either on its own. To do so, among metabolite Euclidean distances based on molecular characteristics were weighted by among-metabolite distances derived from the biochemical transformation network. In effect, the combination of these two matrices results from the transformation-based matrix (standardized from 0 to 1) decreasing the characteristics-based distance values. The resulting dendrogram is referred to as the Transformation-Weighted Characteristics Dendrogram (TWCD) (**Supplementary Figure 2e**). Within the TWCD, metabolites that are distinct based on their characteristics but that are close in the transformation network should cluster more closely together

than in the MCD but further apart than in the TD. Such changes can be observed in the TWCD sub-dendrogram in which the O_3S cluster is closer to the low O-content cluster than it was in the MCD (**Supplementary Figure 2f**), while also being further from the single OS_2 compound than it was in the TD. As another example, the low O-content cluster in the TWCD (e.g., metabolites with $O \leq 3$) is further from the other CHO-metabolites than it was in either the MCD or TD. This suggests that the molecular characteristics of low O-content metabolites are more different from CHO-metabolites than would be expected given the connecting biochemical transformations between them.

We suggest that by merging molecular characteristics with biochemical transformations, TWCDs carry more information related to functionally relevant attributes of metabolites, relative to MCDs or TDs. Despite merging information from MCDs and TDs, however, analyses performed with TWCDs will not be as sensitive to differences in either molecular characteristics or the biochemical transformation network. This again indicates an opportunity to draw out deeper insights by combining patterns derived from multiple dendrograms. We therefore suggest that while there are other approaches to estimating dendrograms from metabolite data, the MCD, TD, and TWCD provide a complementary set of analysis tools that are useful for studying the spatiotemporal organization of metabolite assemblages.

Supplementary References

1. Kim S, Kramer RW, Hatcher PG. Graphical method for analysis of ultrahigh-resolution broadband mass spectra of natural organic matter, the van Krevelen diagram. Anal Chem. 2003;75: 5336–44. Available: http://www.ncbi.nlm.nih.gov/pubmed/14710810
2. Bailey VL, Smith AP, Tfaily M, Fansler SJ, Bond-Lamberty B. Differences in soluble organic carbon chemistry in pore waters sampled from different pore size domains. Soil Biol Biochem. Elsevier Ltd; 2017;107: 133–143. doi:10.1016/j.soilbio.2016.11.025
3. Rivas-Ubach A, Liu Y, Bianchi TS, Tolić N, Jansson C, Paša-Tolić L. Moving beyond the van Krevelen Diagram: A New Stoichiometric Approach for Compound Classification in Organisms. Anal Chem. 2018;90: 6152–6160. doi:10.1021/acs.analchem.8b00529
4. Hughey CA, Hendrickson CL, Rodgers RP, Marshall AG, Qian K. Kendrick Mass Defect Spectrum: A Compact Visual Analysis for Ultrahigh-Resolution Broadband Mass Spectra. Anal Chem. 2001;73: 4676–4681. doi:10.1021/ac010560w
5. Koch BP, Dittmar T. From mass to structure: an aromaticity index for high-resolution mass data of natural organic matter. Rapid Commun Mass Spectrom. 2006;20: 926–932. doi:10.1002/rcm.2386
6. LaRowe DE, Van Cappellen P. Degradation of natural organic matter: A thermodynamic analysis. Geochim Cosmochim Acta. Elsevier Ltd; 2011;75: 2030–2042. doi:10.1016/j.gca.2011.01.020
7. Tfaily MM, Chu RK, Tolić N, Roscioli KM, Anderton CR, Paša-Tolić L, et al. Advanced solvent based methods for molecular characterization of soil organic matter by high-resolution mass spectrometry. Anal Chem. 2015;87: 5206–5215. doi:10.1021/acs.analchem.5b00116
8. Boye K, Noël V, Tfaily MM, Bone SE, Williams KH, Bargar JR, et al. Thermodynamically controlled preservation of organic carbon in floodplains. Nat Geosci. 2017;10: 415–419. doi:10.1038/ngeo2940
9. Dalcin Martins P, Hoyt DW, Bansal S, Mills CT, Tfaily M, Tangen BA, et al. Abundant

carbon substrates drive extremely high sulfate reduction rates and methane fluxes in Prairie Pothole Wetlands. 2017; 3107–3120. doi:10.1111/gcb.13633
10. Tolić N, Liu Y, Liyu A, Shen Y, Tfaily MM, Kujawinski EB, et al. Formularity: Software for Automated Formula Assignment of Natural and Other Organic Matter from Ultrahigh-Resolution Mass Spectra. Anal Chem. 2017;89: 12659–12665. doi:10.1021/acs.analchem.7b03318
11. Graham EB, Tfaily MM, Crump AR, Goldman AE, Bramer LM, Arntzen E, et al. Carbon Inputs From Riparian Vegetation Limit Oxidation of Physically Bound Organic Carbon Via Biochemical and Thermodynamic Processes. J Geophys Res Biogeosciences. 2017;122: 3188–3205. doi:10.1002/2017JG003967
12. Graham EB, Crump AR, Kennedy DW, Arntzen E, Fansler S, Purvine SO, et al. Multi 'omics comparison reveals metabolome biochemistry, not microbiome composition or gene expression, corresponds to elevated biogeochemical function in the hyporheic zone. Sci Total Environ. Elsevier B.V.; 2018;642: 742–753. doi:10.1016/j.scitotenv.2018.05.256
13. Stegen JC. At the Nexus of History, Ecology, and Hydrobiogeochemistry: Improved Predictions across Scales through Integration. mSystems. 2018;3: e00167-17. doi:10.1128/mSystems.00167-17
14. D'Atri V, Causon T, Hernandez-Alba O, Mutabazi A, Veuthey J-L, Cianferani S, et al. Adding a new separation dimension to MS and LC-MS: What is the utility of ion mobility spectrometry? J Sep Sci. 2018;41: 20–67. doi:10.1002/jssc.201700919
15. Fu Q-L, Fujii M, Riedel T. Development and comparison of formula assignment algorithms for ultrahigh-resolution mass spectra of natural organic matter. Anal Chim Acta. 2020;1125: 247–257. doi:10.1016/j.aca.2020.05.048
16. Breitling R, Ritchie S, Goodenowe D, Stewart ML, Barrett MP. Ab initio prediction of metabolic networks using Fourier transform mass spectrometry data. Metabolomics. 2006;2: 155–164. doi:10.1007/s11306-006-0029-z
17. Moritz F, Kaling M, Schnitzler J, Schmitt-Kopplin P. Characterization of poplar metabotypes via mass difference enrichment analysis. Plant Cell Environ. 2017;40: 1057–1073. doi:10.1111/pce.12878
18. Stegen JC, Johnson T, Fredrickson JK, Wilkins MJ, Konopka AE, Nelson WC, et al. Influences of organic carbon speciation on hyporheic corridor biogeochemistry and microbial ecology. Nat Commun. Springer US; 2018;9: 585. doi:10.1038/s41467-018-02922-9
19. Hug LA, Castelle CJ, Wrighton KC, Thomas BC, Sharon I, Frischkorn KR, et al. Community genomic analyses constrain the distribution of metabolic traits across the Chloroflexi phylum and indicate roles in sediment carbon cycling. Microbiome. 2013;1: 22. doi:10.1186/2049-2618-1-22
20. Parks DH, Rinke C, Chuvochina M, Chaumeil P-A, Woodcroft BJ, Evans PN, et al. Recovery of nearly 8,000 metagenome-assembled genomes substantially expands the tree of life. Nat Microbiol. Springer US; 2017;903: 1–10. doi:10.1038/s41564-017-0012-7
21. Parks DH, Chuvochina M, Waite DW, Rinke C, Skarshewski A, Chaumeil P-A, et al. A standardized bacterial taxonomy based on genome phylogeny substantially revises the tree of life. Nat Biotechnol. 2018;36: 996–1004. doi:10.1038/nbt.4229

Supplementary Table 1: A table providing coordinates for the field sites analyzed in this manuscript.

Site	Latitude	Longitude
**A**	46.376667	-119.27293
**B**	46.376333	-119.27292
**C**	46.375397	-119.27266
**D**	46.372975	-119.27208
**E**	46.371753	-119.2716

Supplementary Figure 1: Spatial distribution of sampled field sites in southeast Washington State, including photos of surficial sediments at each sampling location. The quadrant is 1m x 1m. Three independent push point samplers were used in each quadrant to collect pore water and a river water sample was collected from an adjacent location.

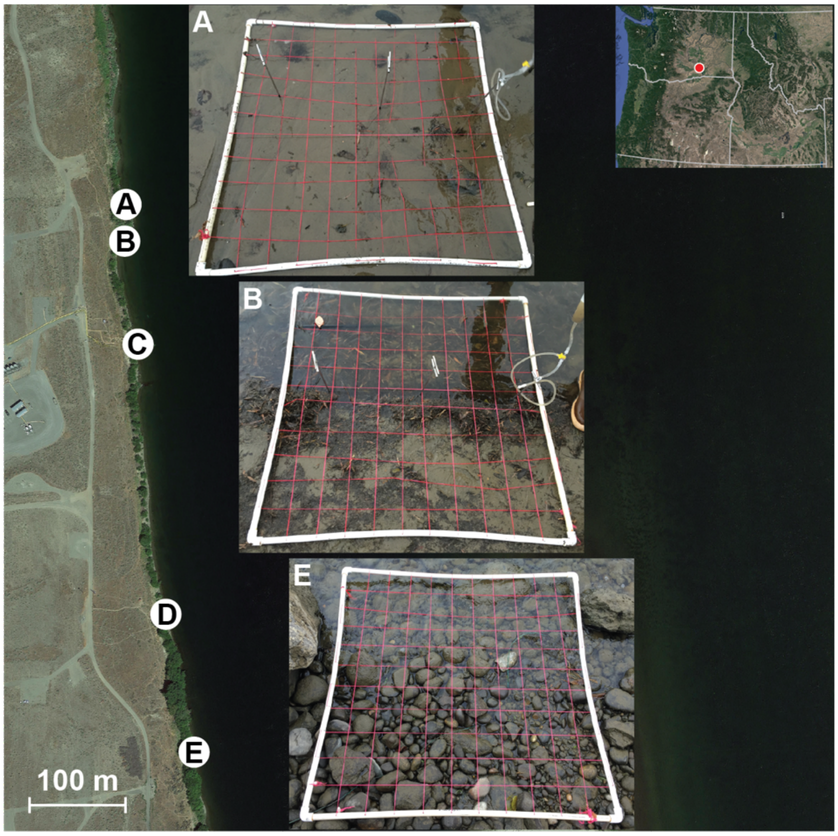

Supplementary Figure 2: Dendrograms and sub-dendrograms with associated metrics. Panels a, c, and e are the visualizations of the Molecular Characteristics Dendrogram (MCD), Transformation-based Dendrogram (TD), and Transformation-Weighted Characteristics Dendrogram (TWCD), respectively. Panels b, d, and f contain sub-dendrograms of the 16 most frequently observed metabolites to demonstrate the clustering patterns of elemental composition and two metrics: average transformation count and nominal oxidation state of carbon (NOSC). Average transformation count is the average number of transformations associated with a given

metabolite across all of the samples. NOSC is a metric that represents the thermodynamic availability of metabolites.

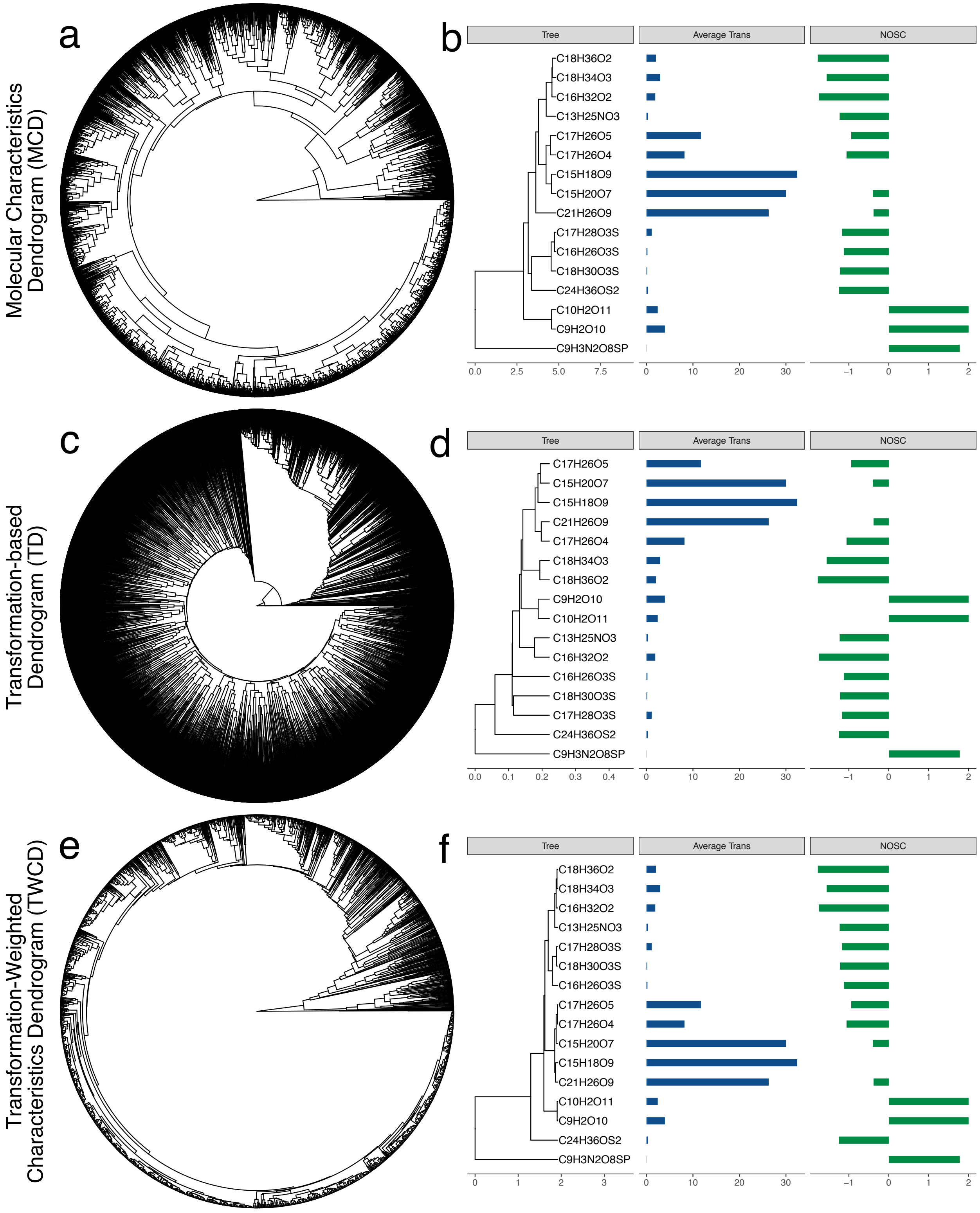

Supplementary Figure 3: A bar chart depicting the number of FTICR-MS detected peaks obtained across the dataset (Total), the number peaks assigned a molecular formula (Assigned), and the number of peaks incorporated into each of the three dendrograms (MCD, TD, TWCD). The numbers above each bar correspond to the height of the bar.

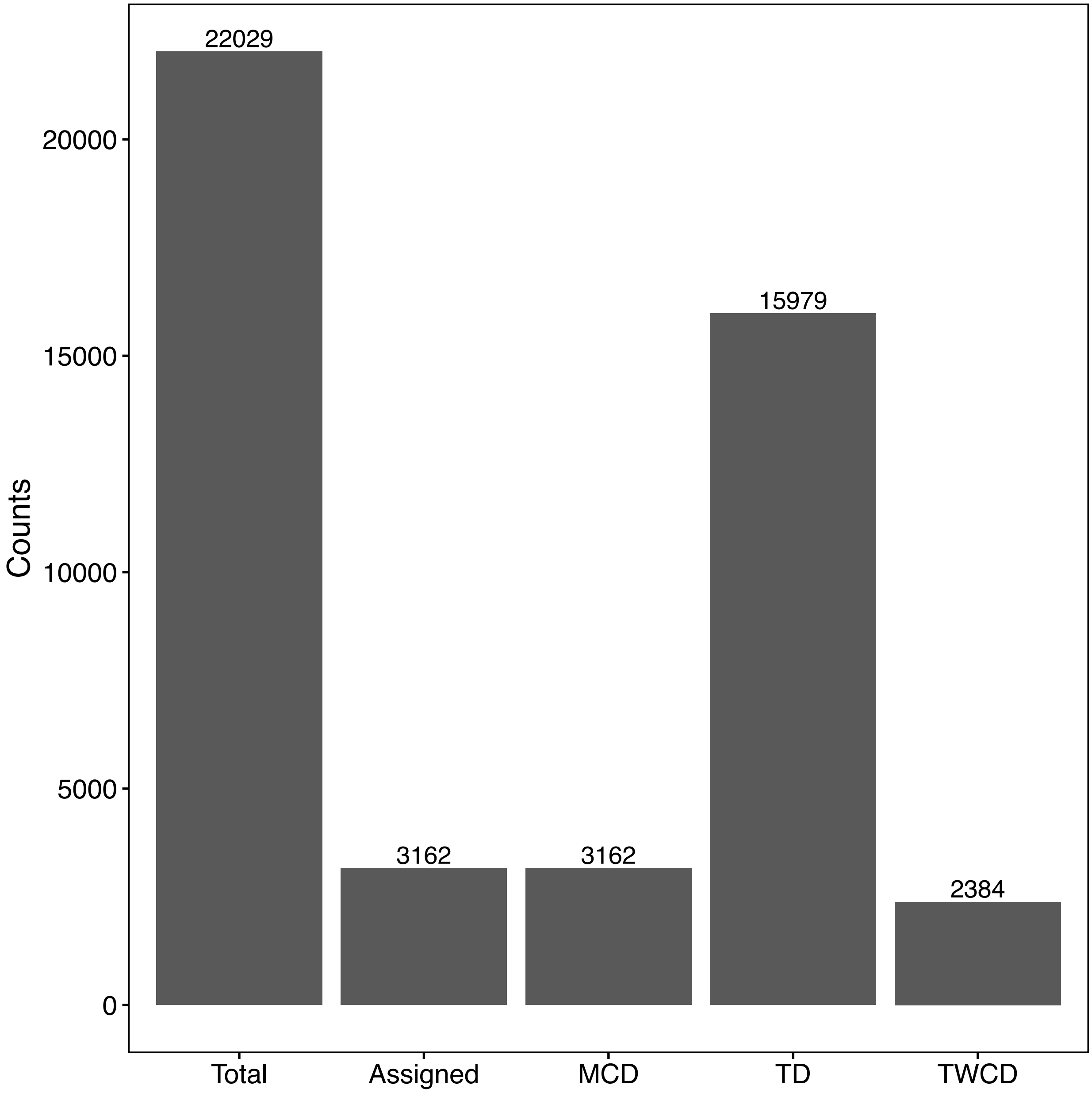

Supplementary Figure 4: Histogram demonstrating the number of FTICR-MS identified peaks with a given number of associated biochemical transformations. The inset plots only those peaks with 100 or fewer transformation counts; the arrow indicates the location of the 40 count which only had 1.

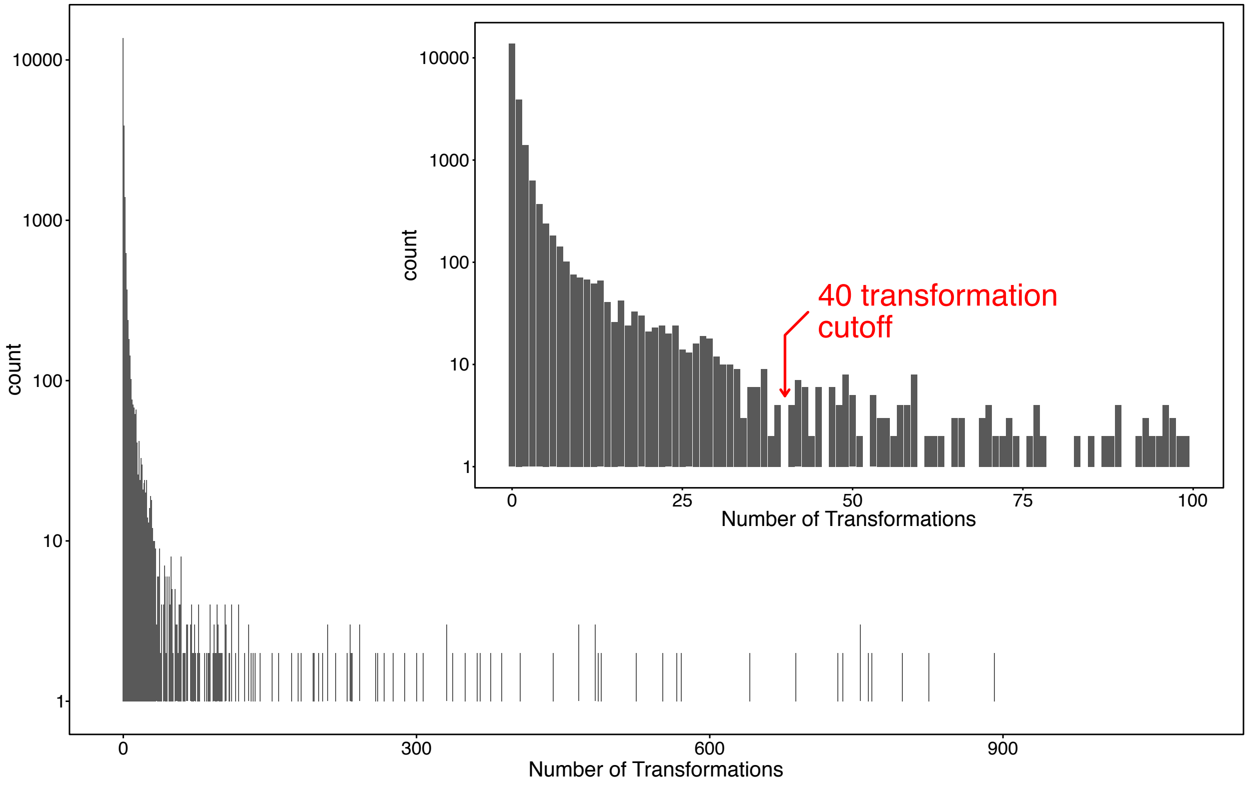

Supplement: Supplementary file 1 — Supplementary Information [file 41467_2020_19989_MOESM1_ESM.pdf]
