# Supplementary material for: Using metacommunity ecology to understand environmental metabolomes
Source: Nat Commun. 2020 Dec 11;11:6369. doi: 10.1038/s41467-020-19989-y (PMC7732844; doi:10.1038/s41467-020-19989-y)
Supplement: Supplementary file 3 — Description of Additional Supplementary Files [file 41467_2020_19989_MOESM3_ESM.pdf]

Description of Additional Supplementary Files

File Name: Supplementary Data 1
Description: Molecular characteristics dendrogram (MCD) generated using the UPGMA hierarchical clustering method.

File Name: Supplementary Data 2
Description: Transformationbased dendrogram (TD) generated using the UPGMA hierarchical clustering method.

File Name: Supplementary Data 3
Description: Transformationweighted characteristics dendrogram (TWCD) generated using the UPGMA hierarchical clustering method.

File Name: Supplementary Data 4
Description: The file is the database of transformations used in the transformation analysis. The first column represents the transformation label, while the second column is the corresponding mass difference. There are two types of transformations listed in this file: 1) the gain or loss of the listed molecular formula (e.g., C1H1O1N1) with numeric values indicating the number of atoms associated with the element that precedes the numeric value, and 2) a substitution reaction denoted by an underscore (e.g., C1H1N1O_1). In the case of a substitution reaction the underscore connects the element lost to the number of atoms lost. For example, C1H1N1O_1 indicates a molecule gained C1H1N1 and lost one O atom. Some substitution reactions include multiple elements that are lost such that there are multiple underscores. In all cases, an underscore connects the element lost to the number of atoms lost. In all cases, atoms are gained if they are not followed immediately by an underscore. For example, C_1H_4O2 indicates loss of one C, loss of four H, and gain of two O. If no numeric value follows an element, it indicates there is a gain of a single atom of that element (e.g., CH2 indicates one atom of C).
