# Supplementary material for: Using metacommunity ecology to understand environmental metabolomes
Source: Nat Commun. 2020 Dec 11;11:6369. doi: 10.1038/s41467-020-19989-y (PMC7732844; doi:10.1038/s41467-020-19989-y)
Supplement: Supplementary file 8 — Reporting Summary [file 41467_2020_19989_MOESM8_ESM.pdf]

nature research

Corresponding author(s): James C. Stegen
Last updated by author(s): Oct 12, 2020

Reporting Summary

Nature Research wishes to improve the reproducibility of the work that we publish. This form provides structure for consistency and transparency in reporting. For further information on Nature Research policies, see Authors & Referees and the Editorial Policy Checklist .

Statistics

For all statistical analyses, confirm that the following items are present in the figure legend, table legend, main text, or Methods section.

n/a	Confirmed	
☐	☒	The exact sample size (n) for each experimental group/condition, given as a discrete number and unit of measurement
☒	☐	A statement on whether measurements were taken from distinct samples or whether the same sample was measured repeatedly
☐	☒	The statistical test(s) used AND whether they are one- or two-sided<br>*Only common tests should be described solely by name; describe more complex techniques in the Methods section.*
☒	☐	A description of all covariates tested
☒	☐	A description of any assumptions or corrections, such as tests of normality and adjustment for multiple comparisons
☒	☐	A full description of the statistical parameters including central tendency (e.g. means) or other basic estimates (e.g. regression coefficient) AND variation (e.g. standard deviation) or associated estimates of uncertainty (e.g. confidence intervals)
☐	☒	For null hypothesis testing, the test statistic (e.g. F, t, r) with confidence intervals, effect sizes, degrees of freedom and P value noted<br>*Give P values as exact values whenever suitable.*
☒	☐	For Bayesian analysis, information on the choice of priors and Markov chain Monte Carlo settings
☒	☐	For hierarchical and complex designs, identification of the appropriate level for tests and full reporting of outcomes
☐	☒	Estimates of effect sizes (e.g. Cohen's d, Pearson's r), indicating how they were calculated

Our web collection on statistics for biologists contains articles on many of the points above.

Software and code

Policy information about availability of computer code

Data collection	No software was used to collect data.
Data analysis	FTICR-MS data was processed using Bruker DataAnalysis (v4.2), Formularity (Tolic et al., 2017 - Anal. Chem.), ftmsRanalysis (Bramer et al., 2020 - PLOS Comp. Bio.), and various R scripts (v3.5.1) which have been deposited on GitHub (https://github.com/danczakre/Meta-Metabolome_Ecology)

For manuscripts utilizing custom algorithms or software that are central to the research but not yet described in published literature, software must be made available to editors/reviewers. We strongly encourage code deposition in a community repository (e.g. GitHub). See the Nature Research guidelines for submitting code & software for further information.

Data

Policy information about availability of data

All manuscripts must include a data availability statement. This statement should provide the following information, where applicable:

- Accession codes, unique identifiers, or web links for publicly available datasets
- A list of figures that have associated raw data
- A description of any restrictions on data availability

Microbial 16S rRNA gene sequencing data is accessible from NCBI via the Bioproject number PRJNA576070, Biosample numbers SAMN16450882 through SAMN16450904. Peak-picked, unaligned FTICR-MS data is accessible on ESS-DIVE at https://data.ess-dive.lbl.gov/view/doi:10.15485/1675028110. The aligned FTICR-MS data report used in this study is available on GitHub at https://github.com/danczakre/Meta-Metabolome_Ecology.

Field-specific reporting

Please select the one below that is the best fit for your research. If you are not sure, read the appropriate sections before making your selection.

- [ ] Life sciences
- [ ] Behavioural & social sciences
- [x] Ecological, evolutionary & environmental sciences

For a reference copy of the document with all sections, see nature.com/documents/nr-reporting-summary-flat.pdf

Ecological, evolutionary & environmental sciences study design

All studies must disclose on these points even when the disclosure is negative.

Study description	This study set out to understand the deterministic and stochastic processes affecting the structure of metabolite assemblies. By comparing the assembly patterns experienced within the surface and pore water, we were able to lay the foundations for a new conceptual synthesis termed "meta-metabolome ecology".
Research sample	Samples consisted of surface river water and pore water (i.e., the hyporheic zone).
Sampling strategy	At each location, one replicate of river water was collected, and 3 pore water samples were collected and filtered using a 0.2 µm Sterivex filters. Pore water replicates were collected from 30cm depth within a 1m^2 area using 0.25-inch diameter sampling tubes. No statistics were done to determine sample sizes and specific sample sizes were not necessary because this study is purely exploratory.
Data collection	Metabolite assemblage data was obtained using Fourier Transform Ion Cyclotron Resonance mass spectrometry (FTICR-MS). Microbial community data was collected using an Illumina MiSeq the V4 region of 16S rRNA genes was amplified and sequenced using the universal bacterial/archaeal primer set 515F/806R.
Timing and spatial scale	Samples were collection across the primary sediment textures found within the study site. This was meant to maximize variation in sampled organic matter chemistry and microbial communities for use as demonstration data sets. The sampling occurred once on each of two separate days, 17th and 19th November 2017. The spatial and temporal extent/frequency were selected to generate enough data from enough locations to enable use of the data as a demonstration of analytical tools. The spatial and temporal extent/frequency, and number of samples, were not designed to enable robust statistical analyses tied to a specific hypothesis.
Data exclusions	Data were not excluded from this study.
Reproducibility	We have provided all the data and code that we to analyze the data to ensure other researchers and reproduce, augment, and confirm (or deny) our results.
Randomization	Samples to be run on the FTICR-MS were randomized prior to data collection in order to limit instrumentation bias.
Blinding	Blinding is not relevant to microbial communities or dissolved organic matter.

Did the study involve field work? [x] Yes [ ] No

Field work, collection and transport

Field conditions	The study was conducted along the Columbia River in Washington, USA. All samples are aquatic and water temperatures ranged between 10-11C during sampling.
Location	Samples were collected along shore of the Columbia River, nearby to Richland, WA, at approximately the following coordinates: 46.372315392331 N 119.26906405851 W
Access and import/export	Samples were processed locally relative to collection location such that were no import/export considerations. Samples were collected in compliance the following permitting: Washington Department of Natural Resources Aquatic Lands Right of Entry (#23-A91879), U.S. Fish and Wildlife Service Endangered Species Act (#01EWFW00-2018-I-0489), and the Cultural Compliance Review (#2015-PNSO-011, #2015-PNSO-011a, & HCRC#2013-300-005b).
Disturbance	Pore water samples were collected 30 cm long, 0.25 inch sampling tubes that have a negligible impact to the field system.

Reporting for specific materials, systems and methods

We require information from authors about some types of materials, experimental systems and methods used in many studies. Here, indicate whether each material, system or method listed is relevant to your study. If you are not sure if a list item applies to your research, read the appropriate section before selecting a response.

Materials & experimental systems

n/a	Involved in the study
☒	☐ Antibodies
☒	☐ Eukaryotic cell lines
☒	☐ Palaeontology
☒	☐ Animals and other organisms
☒	☐ Human research participants
☒	☐ Clinical data

Methods

n/a	Involved in the study
☒	☐ ChIP-seq
☒	☐ Flow cytometry
☒	☐ MRI-based neuroimaging
